# Bacteremia caused by *Bergeyella zoohelcum* in an infective endocarditis patient: case report and review of literature

**DOI:** 10.1186/s12879-017-2391-z

**Published:** 2017-04-12

**Authors:** Yili Chen, Kang Liao, Lu Ai, Penghao Guo, Han Huang, Zhongwen Wu, Min Liu

**Affiliations:** Department of Laboratory Medicine, The First Affiliated Hospital of Sun Yat-sen University, Guangzhou, Guangdong 510080 China

**Keywords:** *Bergeyella zoohelcum*, Bacteremia, Infective endocarditis

## Abstract

**Background:**

*Bergeyella zoohelcum* is an aerobic, Gram-negative bacterium that is frequently isolated from the upper respiratory tract of dogs, cats and other mammals. Clinically, *B. zoohelcum* has been reported causing cellulitis, tenosynovitis, leg abscess and septicemia, which is closely connected with animal bites. Here we describe a case of bacteremia in an infective endocarditis (IE) patient caused by *B. zoohelcum*, in China.

**Case presentation:**

A 27-year-old infective endocarditis woman who had no history of dog bite nor other mammal exposure suffered bacteremia caused by *B. zoohelcum*. This patient, without evidence of polymicrobial infection, was treated with cefuroxime and had a good outcome.

**Conclusions:**

*B. zoolhelcum* bacteremia is rarely reported in IE patients. Our report expands the range of known bacterial causes of infective endocarditis.

## Background

*Bergeyella* (formerly *Weeksella*) *zoohelcum* is an aerobic, rod-shaped, Gram-negative, non-motile, non-saccharolytic bacterium which is usually isolated from the upper respiratory tract of dogs, cats and other mammals [1–3]. Most reported cases of human infection by these bacteria are due to animal bites or are associated with prolonged exposure to pets [4–7]. Additionally, it is reported that a patient suffered *B. zoohelcum* bacteremia after eating the food prepared with coagulated goat blood [8]. A tsunami victim with cellulitis due to *B. zoohelcum* has been reported as well [9]. Here, we report a case of bacteremia caused by *B. zoohelcum* in an infective endocarditis patient and review of literature.

## Case presentation

A 27-year-old Chinese woman was admitted to hospital (The First Affiliated Hospital of Sun Yat-sen University, Guangzhou, China) due to repeated fever for 3 months, chest distress, tachypnea, and palpitation for a month. The patient has been in her usual state of health before admission. Because of tonsillitis, she got fever and sore throat. She slightly improved after symptomatic treatment but suffered a relapse several days later, with maximum body temperature at 40.3 °C. A detailed history disclosed that the patient did not keep any pets, smoke, drink alcohol, or use illicit drugs. She denied sustaining any dog or cat bites, either. She did not have any recent travel history, either within or outside of China. Moreover, she did not visit farms or any markets with live animals, denying any other potential environmental exposures.

On examination, she had a tympanic temperature of 38.2 °C, blood pressure of 105/43 mmHg, pulse rate of 103/min and respiratory rate of 20/min. Physical examination revealed that 2/6 level systolic murmur was audible at the mitral area, and diastolic murmur was at aortic area. Ultrasonic cardiogram revealed infective endocarditis that the formation of vegetations on the mitral valve with severe mitral insufficiency, aortic valve vegetations with severe aortic insufficiency, and prolapse of anterior tricuspid valve leaflet with moderate tricuspid insufficiency. Laboratory evaluation revealed leukocyte count of 6740/mm3 with 65.5% neutrophils and 23.5% band forms, erythrocyte sedimentation rate of 120 mm/h (normal range, 0-20 mm/h), C-reactive protein of 89 mg/L (normal range, <1 mg/L), brain natriuretic peptide of 702.1 pg/ml (normal range, 0–84.0 pg/ml), and microscopic hematuria.

Blood cultures were obtained on admission day and empiric antibiotic treatment with intravenous drip of cefuroxime 1.5 g every 8 h was started. Both sets of blood cultures were positive after 2 days and isolated a Gram negative rod-shaped bacteria. The organism tested positive for oxidase, catalase, urea and indole; while PYR and DNAase test were negative. Initially, the isolate was misidentified as *Brevundimonas diminuta* by Vitek 2 system with only 87.6% identity (bioMérieux SA, Marcy, France). To confirm the identity of the isolate, a fragment of the 16S rRNA gene was amplified using primer sets 16S–forward (5′- AGA GTT TGA TCC TGG CTC AG-3′) and 16S–reverse (5′- ACG GCT ACC TTG TTA CGA CTT-3′) by the polymerase chain reaction [10] and the resultant polymerase chain reaction product sequenced. The sequencing data were queried against the GenBank 16S rRNA gene database. The best match returned was the *Bergeyella zoohelcum*, ATCC 43767 type strain, with 98.2% identity, whereas the next best match was the *Riemerella anatipestifer,* ATCC 11845 strain, with only 91.2% identity. Although, according to the identity algorithm of the CLSI guideline MM18-A about glucose non-fermenting Gram negative bacilli [11], the 98.2% identity of this *Bergeyella zoohelcum* strain was a bit lower than the requirement of ≥99.0% identity (With >0.8% separation from most closely related species); taken together, the biological characteristics and 16S rRNA gene sequencing data strongly supported the identification of *B. zoohelcum*.

The antibiotic susceptibility of the *Bergeyella zoohelcum* strain was determined by the Kirby-Bauer disk diffusion method on Mueller-Hinton agar plates with 5% horse blood with use of Oxoid disks (Oxoid Ltd., Basingstoke, Hants, RG24 8PW, UK). Although there are no categorical interpretative criteria for antimicrobial susceptibility testing (AST) data for *B. zoohelcum*, the isolate exhibited large inhibition zone (millimeter) for all antimicrobials tested: ampicillin 40 mm, cefixoxime 42 mm, ceftriaxone 38 mm, meropenem 40 mm, erythromycin 26 mm, clindamycin 28 mm, trimethoprim/sulfamethoxazole 40 mm, gentamicin 26 mm, levofloxacin 28 mm, which suggested the *B. zoohelcum* isolate was susceptible to β-lactams and fluoroquinolones. Moreover, the isolate presented negative for the production ofβ-lactamase according to the cefinase disk assay (bioMérieux SA, Marcy, France).

On day 3, the patient showed improvement of the clinical symptoms of fever, chest distress, and tachypnea. On day 10, the patient underwent combination of mitral and aortic valve bioprosthesis replacement, and tricuspid valvoplasty. However, tissue cultures taken from the surgical specimens did not produce notable bacterial growth. The patient completed a 6-week course of treatment with cefuroxime. She was discharged from the hospital in a stable condition and resumed her normal daily activities.

## Discussion

*B. zoohelcum*, formerly belonging to *Weeksella zoohelcum*, is a nonfermentative Gram-negative rod which is a member of the family *Flavobacteriaceae* [12, 13]. It grows well on blood agar; while most strains do not grow on MacConkey’s agar. Colonies are entire, circular, semitransparent, smooth, glossy, butyrous and very sticky, making them hard to remove from solid media. In broth, growth is weak. The colonies show no hemolysis on blood agar plates, while greening occasionally exists around colonies [14].The organisms show positive for oxidase, catalase and indole test, negative for PYR, resistant to colistin, and are non-pigmented [15].

Some strains of *B. zoohelcum* showed fastidious which only grew on chocolate agar and biochemically inactive, thus, preventing them from recovery and identification [8]. Therefore, in this case, the *B. zoohelcum* isolate was misidentified as *Brevundimonas diminuta* by the Vitek 2 system, with only 87.6% identity initially. Similarly, in both of the infective endocarditis cases reported by Kyung Mok Sohn et al. [16], Vitek 2 automated system misidentified the pathogen as *Brevundimonas spp. Brevundimonas spp.*, of which 16S rRNA gene sequences indicated a similarity of 94.9% with *Bergeyella zoohelcum*, suggesting that they belonged to a new species of *Bergeyella*. Notably, it was reported by Jumi Yi et al. [10], that *B. zoohelcum* isolate could be identified by MALDI-TOF MS on the Bruker Biotyper platform (Bruker Daltonics, Billerica, MA) [log(score) value >2.0], due to its competency of detecting the species-specific biomarkers instead of an organism’s metabolic activity for identification.

*B. zoohelcum* isolates are proved to be susceptible to penicillin, and resistant to colistin. However, these features are often used for identification rather than antimicrobial susceptibility testing [17]. Though there is no standard antimicrobial agent recommended for the treatment of *B. zoohelcum* infections, it shows highly susceptible to β-lactams and fluoroquinolones. Successful treatment with ampicillin/sulbactam [8], amoxicillin/clavulanic acid, cefazolin with gentamicin, cefuroxime, cefotaxime and ciprofloxacin has been reported [5, 6, 8–10]. In this case, the patient showed improvement of the clinical symptoms of fever, chest tightness, and tachypnea after 3 days of intravenous drip of cefuroxime 1.5 g every 8 h.

*B. zoohelcum* has become an increasingly recognized cause of leg abscess, cellulitis, pneumonia, tenosynovitis, septicemia and meningitis, which is closely related to animal bites [7, 10, 15]. Moreover, worldwide, infective endocarditis caused by *B. zoohelcum* has been reported only in 2 cases ever [16] (Table 1). Although in the present case, the culture from the specimen of the mitral valve vegetation was negative, given that both sets of blood culture isolated *B. zoohelcum,* we assume, this is another rare report on IE caused by *B. zoohelcum.* Notably, most of the cases of *Bergeyella zoohelcum* bacteremia showed the presence of animal bites or mammals exposure (Table 1). *B. zoolhelcum* bacteremia has been previously reported in an 80-year-old diabetic woman who suffered from bed sores and had cat contact [18]. It has also been reported in a 33-year-old man after a dog bite over his left forearm [5]. Kivinen et al. reported that a 77-year-old patient presented with severe skin infection and *B. zoolhelcum* bacteremia after cat contact, who was in poor health taking steroidal and diabetic medication [19]. Beltran et al. reported that a 44-year-old woman got diarrhea and bacteremia, who consumed the food prepared with goat’s blood [8].Wei-Ru lin et al. reported another case of a man who developed *B. zoolhelcum* bacteremia with dog contact history [15]. But recently, Kyung Mok Sohn et al. reported a 26-year-old male and a 47-year-old male who developed *B. zoolhelcum* bacteremia and infective endocarditis without any pets’ exposure [16]. In the present case, the IE patient asserted that she had no history of dog bite nor other mammals exposure. Therefore, in this case, we assumed that dog bites, contact with a cat, or the ingestion of contaminated food may not be the only portal of entry for *B. zoohelcum,* but another unknown route of infection is subject to detection.

**Table 1:** Summary of reported cases of *Bergeyella zoohelcum* infection

Reference	Age (yr)/Sex	Infection	Exposure	Underlying disease	Treatment	Outcome
Noel et al., 1989 [18]	80/F	Bed sore infection	Cat contact	Diabetes mellitus	Cefotaxime	Recovered
Montejo et al., 2001 [5]	33/M	Wound infection	Dog bite	None	Day 1–14: amoxicillin-clavulanic	Recovered
Kivinen et al., 2003 [19]	77/F	Skin infection	Cat contact	Diabetes mellitus, steriod use	Cefotaxime	Recovered
Shukla SK et al., 2004 [7]	60/F	Cellulitis and lymphangitis	Cat bite	Not reported	Day 1: Amoxicillin/clavulanate Day 2–7: Ampicillin/sulbactam	Recovered
Beltran et al., 2006 [8]	44/F	Diarrhea	Goat^’^s blood ingestion	None	Day 2–9: ciprofloxacin	Recovered
Wei-Ru lin et al., 2007 [15]	73/M	Cellulitis	Dog contact	Liver cirrhosis, transitional cell carcinoma of urinary bladder	Day 1–14: Cefazolin and gentamicin	Recovered
Kyung Mok Sohn et al., 2015 [16]	26/M	Infective endocarditis	None	None	Day 4–5:ampicillin/sulbactam Day 6–48:ceftriaxone	Recovered
	47/M	Infective endocarditis	None	Paroxysmal supraventricular tachycardia	Day 1–3: ceftriaxone, ampicillin, and gentamicin Day 4–32: piperacillin/tazobactam and amikacin Day33–36:ampicillin/sulbactam	Recovered
This present case	27/F	Infective endocarditis	None	None	Day1–42:Cefuroxime	Recovered

## Conclusions

In conclusion, *B. zoolhelcum* bacteremia is rarely reported in IE patients. Our report expands the range of known bacterial causes of infective endocarditis. Since, traditional biochemical identification equipment may misidentify such organisms, MALDI-TOF MS and molecular assays will provide more accurate result on identification.

## Abbreviations

B. zoohelcum: Bergeyella zoohelcum
IE: Infective endocarditis
MALDI-TOF MS: Matrix-Assisted Laser Desorption/Ionization Time of Flight Mass Spectrometry
